# Targeting Purinergic Signaling in the Dynamics of Disease Progression in Sepsis

**DOI:** 10.3389/fphar.2020.626484

**Published:** 2021-01-14

**Authors:** Raíssa Leite-Aguiar, Vinícius Santos Alves, Luiz Eduardo Baggio Savio, Robson Coutinho-Silva

**Affiliations:** Laboratory of Immunophysiology, Biophysics Institute Carlos Chagas Filho, Federal University of Rio de Janeiro, Rio de Janeiro, Brazil

**Keywords:** systemic inflammation, CD39, adenosine, immunosuppression, P2X7 receptor

## Introduction

Sepsis is an infection-related syndrome that encompasses distinct conditions in which organ dysfunctions are the main features, resulting from imbalances of host immune responses that can be lethal (Singer et al., 2016). Despite advances in supportive treatments, no specific drug has been explicitly approved for treatment of sepsis. For this reason, sepsis remains a health concern worldwide (Reinhart et al., 2017), accounting for the majority of nosocomial deaths, a statistic that is especially worrisome in developing countries (Rudd et al., 2020). The absence of appropriate treatments still remains a major obstacle.

The pathophysiology of sepsis usually begins with excessive and uncontrolled immune responses to a pathogen with the overlapping secretion of both pro- and anti-inflammatory components, culminating in organ failure (Hotchkiss et al., 2013a; Van Der Poll et al., 2017). Over time, this initial hyperinflammatory state gives way to a dominant hypoinflammatory period (Wiersinga et al., 2014; Van Der Poll et al., 2017). During this second stage, there is a depletion of cytokines, combined with induction of inhibitory signaling molecules and apoptosis or reprogramming of inflammatory cells, resulting in transition to an immunosuppressive state, most recently referred to as a state of circulating leukocyte reprogramming (Boomer et al., 2011; Cavaillon et al., 2020). The host’s mechanisms to dampen excessive inflammation may interfere with the clearance of infectious organisms or may lead to increased host susceptibility to secondary infections, especially by opportunistic pathogens. In addition, the cellular reprogramming may lead to the development of late sequelae in survivors of sepsis (Otto et al., 2011; Hotchkiss et al., 2013b).

Danger-associated molecular patterns (DAMPs), including adenosine triphosphate (ATP), can be released by activated or damaged cells in the extracellular milieu during infectious conditions including sepsis (Cauwels et al., 2014; Idzko et al., 2014; Sumi et al., 2014). Extracellular ATP (eATP) acts as a danger signal molecule (Coutinho-Silva and Ojcius, 2012; Ma et al., 2018), triggering purinergic signaling, which affects immune cell function and influences the initial hyperinflammatory phase of sepsis (Ledderose et al., 2016). Purinergic signaling is a well-conserved system throughout evolution; the pathway includes purinergic receptors, nucleotides, nucleosides, and ectoenzymes called ectonucleotidases that regulate the metabolism of these molecules (Burnstock and Verkhratsky, 2009; Alves et al., 2020). Regarding its composition and ligand affinity, the purinergic receptors are divided into the metabotropic P1 receptors (A_1_, A_2A_, A_2B,_ and A_3_) associated with adenosine (ADO), ionotropic P2X receptors (P2X1-7), and metabotropic P2Y receptors (P2Y_1_, P2Y_2_, P2Y_4_, P2Y_6_, and P2Y_11–14_) for tri- and diphosphonucleotides (Ralevic and Burnstock, 1998; Fredholm et al., 2011; Jacobson et al., 2020). These receptors have distinct roles in inflammatory environments; for example P1 receptors in general can mitigate inflammation and tissue injury, while P2 receptors can stimulate pro-inflammatory responses and promote bacterial killing (Savio et al., 2018; Antonioli et al., 2019; Savio and Coutinho-Silva, 2019). The cleavage of nucleotides occurs mainly through the action of E-NTPDase1/CD39 and ecto-5′-nucleotidase/CD73. The former enzyme catalyzes the hydrolysis of adenosine triphosphate (ATP) and adenosine diphosphate (ADP) into adenosine monophosphate (AMP), and the latter catalyzes the hydrolysis of AMP to ADO (Robson et al., 2006). The purinergic signaling has been studied in the sepsis context. Here, we discuss advances in understanding this signaling in sepsis pathophysiology as well as possible therapeutical interventions based on purinergic components in the phases of sepsis.

## Recent Advances of Purinergic Signaling in Sepsis

In recent years, studies have demonstrated the involvement of purinergic signaling in the pathophysiology of sepsis. ATP exerts a pro-inflammatory response in macrophages, monocytes, and dendritic cells, promoting pro-inflammatory cytokine release (i.e., IL-1β and IL-18) (Grahames et al., 1999; Ferrari et al., 2007), while adenosine stimulates the release of anti-inflammatory cytokines (i.e., IL-10) (Németh et al., 2005). In addition, eATP levels increase neutrophil migration and activation, causing tissue damage and organ injury (Sumi et al., 2014). The P2X7 receptor (P2X7R) has been described as the most relevant purinergic receptor involved in inflammatory processes (Di Virgilio and Pelegrín, 2019). This receptor is widely expressed by immune cells and it mediates the activation of several inflammatory and antimicrobial mechanisms in infection diseases, including sepsis (reviewed in Savio et al., 2018).

P2X7R gain-of-function single nucleotide polymorphisms have been correlated to increase sepsis severity in humans (Geistlinger et al., 2012). A recent study found P2X7R expression is elevated in the surface of monocytes from patients with sepsis. Moreover, cytokine levels (e.g., those of IL-1β, IL-18), the alarmin HMGB1, and ASC aggregates are increased in the serum of these patients (Martínez-García et al., 2019). These findings suggest the involvement of the P2X7-NLRP3 axis in sepsis (Martínez-García et al., 2019). P2X7R pharmacological blockade with BBG decreased levels of inflammatory cytokines (i.e., IL-1β, IL-6, and IL-10), NO production, and neutrophil recruitment to the peritoneal cavity in a mouse model of sepsis. This inhibition decreased liver damage and attenuated activation of inflammatory signaling pathways, demonstrating the protective effect of P2X7R inhibition in the initial phase of sepsis (Savio et al., 2017b). Similarly, genetic deletion of P2X7R or treatment with the antagonist A438079 decreased the mortality rate in sepsis induced by cecal ligation and puncture (CLP) (Santana et al., 2015; Wang et al., 2015). Corroborating these data, P2X7R activation by BzATP promoted excessive inflammation and disruption of the intestinal barrier, while systemic blockade using P2X7 antagonist A740003 protected mice against sepsis (Wu et al., 2017). P2X7R is also directly connected to oxidative stress and pro-inflammatory cytokines secretion in the liver (Larrouyet-Sarto et al., 2020) and brain of septic mice (Savio et al., 2017a). Interestingly, these effects are tightly restrained by CD39 activity (Savio et al., 2017a).

Di Virgilio and Pelegrín reported that, even though the P2X7R has been described as the purinergic receptor most involved in inflammatory processes, recent findings suggest that P2X4 receptor (P2X4R) exhibits relevant contributions in this context as well (Di Virgilio and Pelegrín, 2019). Csoka and colleagues showed that ATP is responsible for *Escherichia coli* and *Staphylococcus aureus* killing in wild-type macrophages, and this effect is independent of P2X7Rs (Csóka et al., 2018). Using CD39^−/−^ mice, they demonstrated that adenosine was not responsible for bacterial killing. In addition, they showed that ATP failed to destroy these pathogens in macrophages isolated from P2X4^−/−^ mice. P2X4 expression levels were elevated in liver and lung of septic mice. By contrast, in peritoneal monocytes/macrophages and neutrophils, expression levels were decreased, suggesting that, in the CLP model, P2X4R has a protective role (Csóka et al., 2018).

Another purinergic receptor that may have a role in sepsis pathogenesis is P2X1 (P2X1R). In a model of urosepsis using an *E. coli* strain, the inhibition of this receptor with two different antagonists (NF279 and NF449) showed that this receptor could not protect the host against sepsis. P2X1R antagonism promoted an increased pro-inflammatory cytokine release (i.e., IL-1β, TNF-α, and IL-6) and higher bacterial load, decreasing survival in mice (Skals et al., 2019).

Despite the importance of P2X receptors, P2Y receptors can also be relevant in sepsis. Interestingly, a recent report demonstrated that eATP increased in the peritoneal cavity and systemic circulation of mice subjected to the CLP model. This increase was confirmed using LPS-primed peritoneal macrophages that showed a connexin-43-dependent pathway for ATP release. This nucleotide acts through in autocrine manner, activating P2Y_1_ receptor and then inducing the release of pro-inflammatory cytokines (Dosch et al., 2019). Accordingly, LPS-stimulated monocytes release ATP that can suppress T cell responses. eATP can activate the P2Y_11_ receptor, which impairs mitochondrial activity and blocks T cell migration required for host defense in sepsis (Sueyoshi et al., 2019). Furthermore, the P2Y_12_ antagonist (clopidogrel) reduced the number of white blood cells (WBCs), including lymphocytes and neutrophils in septic mice. Clopidogrel also significantly reduced sepsis-induced lung injury. P2Y_12_ receptor-deficient mice also showed diminished production of inflammatory mediators (i.e., IL-6, TNF-α, IL-10, and MIP-1) and reduced sepsis-induced lung injury (Liverani et al., 2016).

An important mechanism that can protect against sepsis are the activities of the ectoenzymes E-NTPDase1/CD39 and ecto-5′-nucleotidase/CD73, which are responsible for catalyzing the degradation of ATP to adenosine. Ectonucleotidase activities increased in lymphocytes and macrophages from septic mice (Vuaden et al., 2011; Savio et al., 2017b). CD39 diminished the inflammation and enhanced the survival of septic mice due to its ability to scavenge eATP (Csóka et al., 2015). CD39 is essential to limit P2X7R pro-inflammatory effects in sepsis (Csóka et al., 2015; Savio et al., 2017b). Moreover, CD39 overexpression inhibited the NLRP3 inflammasome activation, which decreased inflammation and mitigated sepsis-induced organ injury (Yang et al., 2019). CD73 deficiency also decreased survival, bacterial clearance, and increased cytokine and chemokine production in CLP-induced sepsis (Haskó et al., 2011). These reports demonstrate the crucial role of these enzymes in protecting against inflammation and host organ injury in the initial stages of sepsis, because they promote adenosine formation in the extracellular milieu. However, adenosine generated by these enzymes may contribute to cellular reprogramming and development of immunosuppression in the latter stages of sepsis.

Adenosine receptors have also been studied in sepsis, especially A_2A_ and A_2B_ (Rehman et al., 2020). In a mouse model of endotoxemia, pharmacological activation of A_2A_ receptor improved survival rates and reduced bacteremia (Sullivan et al., 2004). A similar protective profile in septic mice treated with A_2A_ agonists was observed when mice were infected with gram-positive and gram-negative bacteria, including an increase of anti-inflammatory and decreased pro-inflammatory cytokines (Moore et al., 2008). Nevertheless, A_2A_ stimulation can be ambiguous in a polymicrobial infection. In a peritonitis model caused by the injection of a fecal solution, survival was higher and bacterial load was lower in A_2A_-deficient animals (Meriño et al., 2020). In CLP-induced sepsis, A_2A_ knockout or antagonism likewise enhanced survival, in addition to attenuating anti-inflammatory cytokines levels and bacterial burden in serum and peritoneal lavage fluid (Németh et al., 2008). Conflicting results were obtained in studies regarding the A_2B_ receptor in sepsis. Genetic deletion or pharmacological blockade decreased mortality rates by increasing active macrophage phagocytosis and bacterial clearance (Belikoff et al., 2011). By contrast, another study using the same polymicrobial infection model and the same approaches, including the antagonist used, resulted in a higher mortality rate (Csóka et al., 2010).

Interestingly, combined approaches appear to be beneficial in the initial stages of CLP-induced sepsis. Combined A_2A_ activation and P2X7 inhibition decreased hepatic cell death liver injury, demonstrating the relevance of CD39 activity for restricting pro-inflammatory mechanisms and providing substrates for CD73, thereby providing adenosine in the extracellular milieu (Savio et al., 2017b). Indeed, in a study regarding septic cardiomyopathy, septic mice showed diminished ischemia and reperfusion injury, presumably mediated by upregulation of both A_2A_ and A_2B_ expression in ventricles, as their blockade essentially abolished this cardioprotective effect (Busse et al., 2016). Finally, A_1_ receptor antagonism, genetic ablation, and desensitization were all associated with lymphopenia, a clinical feature of sepsis that correlates with more significant lethality (Riff et al., 2017).

## Conclusion and Future Directions

Sepsis is a complex and uncontrolled systemic inflammation caused by pathogen infection. Usually, sepsis is caused by bacteria; nevertheless, some viruses can also induce systemic inflammatory responses, including the recently described severe acute respiratory syndrome coronavirus 2 (SARS-Cov2) (Li et al., 2020). Interestingly, purinergic signaling is also potentially involved in the pathogenesis of SARS-Cov2, considering its role in IL-1β secretion (Di Virgilio et al., 2020). Recently, Huet and colleagues showed that a human IL-1 receptor antagonist (anakinra) improved outcomes and decreased mortality among patients with severe forms of SARS-Cov2 (Huet et al., 2020).

Sepsis is a dynamic syndrome that can be divided into two phases. The first one is commonly known as an intense inflammatory phase, and the second is associated with an immunosuppressive state, which refers to lymphocyte exhaustion and immune cell reprogramming. Considering the high costs to the health systems, the difficulty of managing sepsis, and the consequences for patients who survive and develop long-term sequelae, it is imperative to identify new therapies to improve these outcomes. Therefore, even though antibiotic treatment is the primary approach in sepsis, new procedures are necessary to prevent adjacent immune abnormalities caused by this disease. Interventions targeting purinergic signaling components could be interesting adjuvant therapies.

The severity and the phases of sepsis should be considered to develop therapeutical strategies based on purinergic signaling. According to the studies discussed here, P2 receptors, mainly P2X4 and P2X7 receptors, were able to activate microbicidal mechanisms and induce pro-inflammatory cytokines release, which can be necessary for pathogen control, but at the same time can be related to the initial hyperinflammatory phase of sepsis, causing organ dysfunction and poor outcomes. On the other hand, adenosine, acting mainly via A_2A_ and A_2B_ receptors, may promote anti-inflammatory cytokines release and attenuate tissue injury, suggesting a protective role in initial sepsis phases. Nevertheless, adenosine-based interventions should be carefully analyzed. This molecule can contribute to the reprogramming of immune cells to an immunosuppressive phenotype, causing secondary infections and long-term sequelae.

**FIGURE 1:**
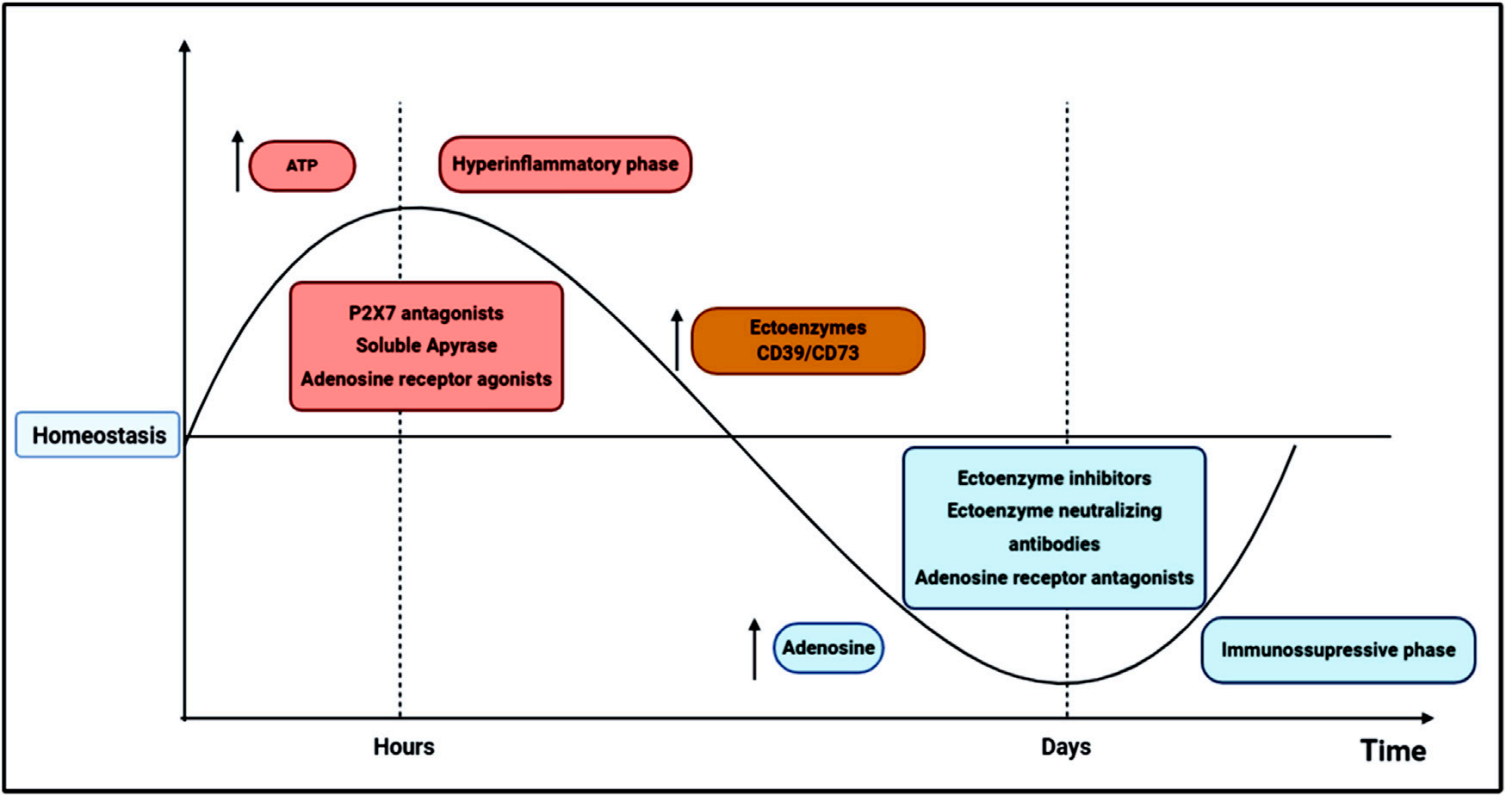
Schematic representation of possible therapeutic interventions based on purinergic signaling components in sepsis dynamics. In the first 24–72 h, sepsis pathogenesis is characterized by excessive innate and adaptive immune responses, in which high levels of cytokines and other inflammatory mediators are produced, inducing organ dysfunction and ultimately, a high mortality rate. This initial hyperinflammatory phase (in red) can be modulated by ATP secretion from activated and damaged cells. ATP activates P2 receptors involved in pro-inflammatory reactions, such as the P2X7 receptor, which has been related to poor outcomes in sepsis. Thus, the administration of P2X7 antagonists has been described as a potential therapeutic target in the initial phase sepsis, reducing inflammatory cytokines release, tissue damage and mortality. Adenosine is commonly associated with inflammation control and tissue protection; therefore, adenosine agonists and soluble ectoenzymes may also represent interesting therapeutic strategies to control the initial hyperinflammatory phase. After some days of disease progression, a secondary state arises where a diminished cytokine secretion is verified, and inflammatory cells are directed to an apoptotic or reprogrammed state, which in turn may lead to an immunosuppressive phase (in blue), where opportunistic infections are most likely to occur increasing mortality. In the transition from hyper-to a hypoinflammatory phase, ectoenzyme activities, and adenosine availability increase in the extracellular milieu, possibly favoring cellular reprogramming and immunosuppression development. Therefore, in the second stage of sepsis, P1 receptor antagonists and CD39/CD73 neutralizing antibodies or inhibitors could restrain the immunosuppressive state, reducing host susceptibility to secondary infections and late metabolic and immune alterations.

A limiting mechanism in this context is the functionality of ectoenzymes CD39/CD73 that are essential for the degradation of ATP into adenosine, contributing to the switch between pro-inflammatory and anti-inflammatory responses in sepsis (Figure 1). Therefore, the CD39/CD73 axis appears to be protective in the initial phase of sepsis, reducing the excessive inflammation. Nevertheless, the increased expression of these enzymes by immune cells and the continuous adenosine generation during the disease progression may also contribute to immunosuppression and late sequelae.

Therefore, the use of P2 receptor antagonists and soluble apyrases may be an attractive therapeutic approach in association with antibiotics to dampen excessive inflammation and control infection in the initial phase of sepsis. In addition, natural polyphenolic compounds have shown anti-inflammatory properties by inhibiting ATP-P2X7 signaling (Nuka et al., 2018). In the second phase of sepsis, the administration of adenosine antagonists and CD39/CD73 neutralizing antibodies could limit the immunosuppression, reducing the susceptibility to secondary infections and late metabolic and immune alterations (Figure 1). Future studies should consider these observations for the development of adjuvant therapies based on purinergic signaling to manage the immune environment in sepsis.

## Author Contributions

RL-A, and VA drafted the manuscript. LS and RC-S contributed to writing and editing.

## Funding

This work was supported by funds from the Conselho Nacional de Desenvolvimento Cientifico e Tecnológico do Brasil–CNPq (306839/2019-9 to RC-S), Coordenação de Aperfeiçoamento de Pessoal de Nível Superior (CAPES), and Fundação de Amparo à Pesquisa do Estado do Rio de Janeiro-FAPERJ (E-26/202.701/2019 and E-26/010.002260/2019 to LS; 26/010.101036/2018 and E-26/202.774/2018 to RC-S).

## Conflict of Interest

The authors declare that the research was conducted in the absence of any commercial or financial relationships that could be construed as a potential conflict of interest.
